# Parallel veno-venous and veno-arterial extracorporeal membrane circuits for coexisting refractory hypoxemia and cardiovascular failure: a case report

**DOI:** 10.1186/s12871-021-01299-5

**Published:** 2021-03-12

**Authors:** Jose R. Navas-Blanco, Sofia A. Lifgren, Roman Dudaryk, Jeffrey Scott, Matthias Loebe, Ali Ghodsizad

**Affiliations:** 1 Department of Anesthesiology, Perioperative Medicine and Pain Management, University of Miami Miller School of Medicine, Jackson Memorial Hospital, 1800 NW 10 Avenue (M-820), Miami, FL 33136 USA; 2 Department of Critical Care, Miami Transplant Institute, Jackson Memorial Hospital, Miami, FL USA; 3 Department of Surgery, University of Miami Hospital, Miami Transplant Institute, Jackson Memorial Hospital, Miami, FL USA

**Keywords:** Acute respiratory distress syndrome, Intensive care unit, Mechanical ventilation, Extracorporeal membrane oxygenation

## Abstract

**Background:**

The complexity of extracorporeal membrane oxygenation (ECMO) techniques continues to evolve. Different cannulation methods and configurations have been proposed as a response to a challenging cardiovascular and pulmonary physiology of the patients. The use of parallel ECMO circuits represents a unique and novel approach for patients with refractory respiratory failure and cardiovascular collapse with very large body surface areas.

**Case presentation:**

We present the case of a 25-year-old morbidly obese male patient admitted for severe acute respiratory distress syndrome (ARDS) and refractory hypoxemia, requiring institution of double cannulation for veno-venous ECMO. Since his hypoxemia persisted, likely due to insufficient flows given his large body surface area, an additional drainage venous cannula was implemented to provide higher flows, temporarily addressing his oxygenation status. Unfortunately, the patient developed concomitant cardiogenic shock refractory to inotropic support and extracorporeal fluid removal, further worsening his oxygenation status, thus the decision was to institute four-cannulation/parallel-circuits veno-venous and veno-arterial ECMO, successfully controlling both refractory hypoxemia and cardiogenic shock.

**Conclusions:**

Our case illustrates a novel and complex approach for combined severe ARDS and cardiovascular collapse through the use of parallel veno-venous and veno-arterial ECMO circuits, and exemplifies the expansion of ECMO techniques and its life-saving capabilities when conservative approaches are futile.

## Background

The use of Extracorporeal Membrane Oxygenation (ECMO) has been vastly expanded for management of refractory Acute Respiratory Distress Syndrome (ARDS) and respiratory failure in the form of veno-venous (VV) ECMO to provide adequate oxygenation and carbon dioxide removal, and for cardiogenic shock in the form of veno-arterial (VA) to provide partial or complete hemodynamic support [1–3]. For patients with severe respiratory failure on VV-ECMO, the amount of blood flow provided is crucial to assure an adequate oxygenation from the device. In certain pathophysiological circumstances (i.e. high cardiac output states, patients with large body surface area, small ECMO cannulas) the maximal amount of blood flow from the VV-ECMO is not enough to provide adequate oxygenation [4, 5]. Additional ECMO cannulas are therefore needed to overcome this problem, although this may introduce a high blood return that may overwhelm the cardiovascular system and lead to cardiogenic shock [6].

The authors present the case of a 25-year-old severe morbidly obese patient who presented with acute refractory hypoxemic respiratory failure that required double cannulation VV-ECMO. The patient’s oxygenation was still marginal in despite of VV-ECMO, and given his body habitus, an additional drainage cannula was established to provide higher blood flow (VV-V ECMO). Concomitant cardiogenic shock ensued further worsening his respiratory status, therefore the patient was placed on a four cannulation/two-circuit VV and VA ECMO configuration. Both ARDS and cardiogenic shock resolved and the patient was successfully weaned from ECMO and decannulated.

Increased utilization and technological advancements have led to different cannulation techniques. Alternative configurations have been implemented to adapt with the complexity of the ECMO circuit itself or with the clinical context of the patient [7–9]. Informed consent for publication of this case report was obtained from the patient on anticipation of the writing of this manuscript.

## Case presentation

A 25-years-old severe morbidly obese male (Body Mass Index [BMI] 54 kg/m^2^, Body Surface Area [BSA] 3.27m^2^) with no other known past medical history, presented to the emergency department with worsening respiratory distress. Patient’s family denied vaping, electronic cigarette or illicit drug use. On initial evaluation, he was found to have an arterial partial pressure of oxygen (PaO2) of 28 mmHg and an arterial partial pressure of carbon dioxide (PaCO2) of 79 mmHg, non-responding to supplemental oxygen and non-invasive positive pressure ventilation. He was endotracheally intubated, placed on lung protective mechanical ventilation with a fraction of inspired oxygen [FiO2] of 100%, and admitted to the intensive care unit (ICU). Initial respiratory viral panel, blood and respiratory cultures were negative. Chest radiograph demonstrated bilateral pulmonary infiltrates.

**Fig. 1:**
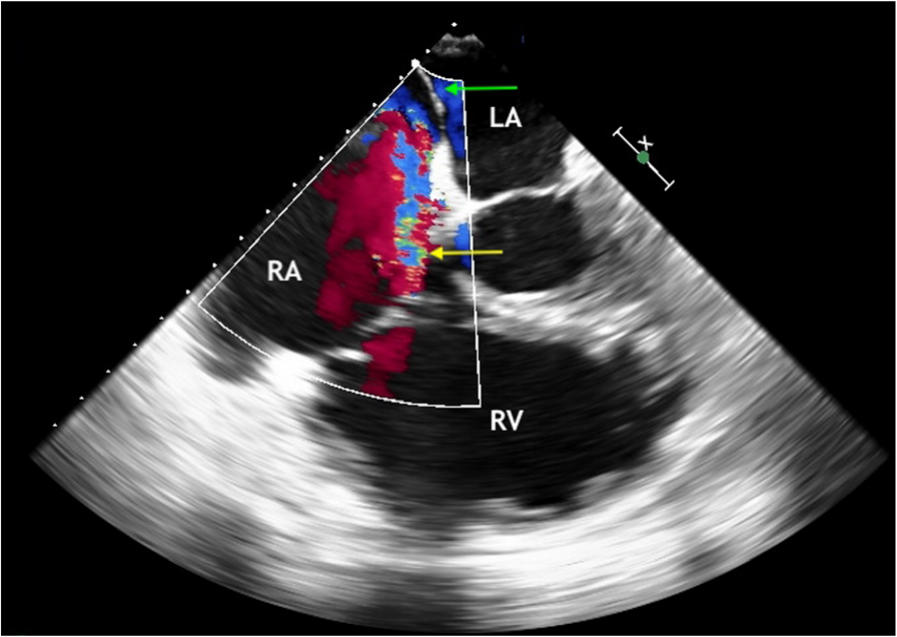
Mid-esophageal four chamber view demonstrating a moderate to severely dilated right ventricle (RV), a posteriorly-directed jet across the tricuspid valve (yellow arrow) into the right atrium (RA), and an inter-atrial septum (green arrow) bowing towards the left atrium (LA)

Initial attempts to evaluate heart function with transthoracic echocardiography were unsuccessful given the patient’s body habitus. Over the following five days, efforts to decrease the fraction inspired on oxygen (FiO2) were unsuccessful (nadir of 80%), and his PaO2/FiO2 ratio ranged around 110-164 mmHg. Pulmonary compliance was still preserved at this point. Next day, the patient developed frank anuria and acute kidney failure, requiring initiation of continuous veno-venous hemodialysis (CVVHD). Transesophageal Echocardiogram (TEE) revealed a mildly dilated left ventricle with moderately reduced function (ejection fraction 30–35%), no signs of wall motion abnormalities, and a moderate-to-severe dilated right ventricle with elevated systolic pressures (50–60 mmHg) (Fig. 1).

The patient hypoxemia worsened (PaO2/FiO2 ratio of 75 mmHg) with associated ventilator desynchrony and worsening pulmonary compliance (plateau pressures 39cmH2O) for which neuromuscular blockade was started. A pulmonary artery catheterization demonstrated elevated pulmonary artery pressures (systolic 58 mmHg, diastolic 32 mmHg, mean 41 mmHg) and a central venous pressure of 14 mmHg. Inotropic support (milrinone) and inhaled epoprostenol were started, all aimed to decrease the pulmonary artery pressures and enhance right ventricular function. In spite of these changes, the patient still remained severely hypoxemic (PaO2 64 mmHg) as well as hypercarbic and acidotic (79 mmHg, pH 7.22, respectively) with normal lactate levels (1.7 mmol/L, normal value < 2 mmol/L), as well as normotensive with no vasopressor requirement.

**Fig. 2:**
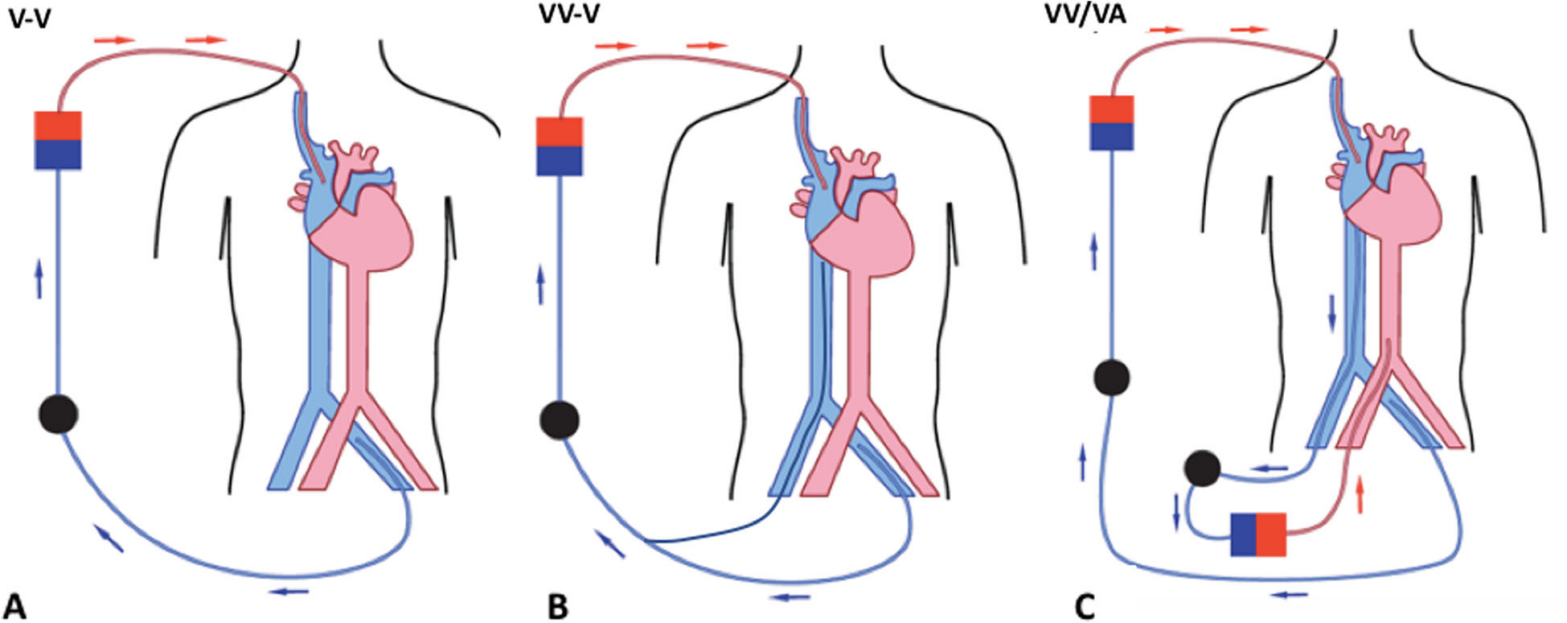
Diagram depicting the progression of the extracorporeal membrane oxygenation (ECMO) cannulation in this patient. **a** Veno-venous (VV) ECMO: drainage cannula in the left femoral vein, return cannula in the right internal jugular vein. **b** Veno-veno-venous ECMO: drainage cannula in the left femoral vein and right femoral vein, return cannula in the right internal jugular vein. **c** Parallel veno-venous and veno-arterial (VA) ECMO. VV: drainage cannula in the left femoral vein, return cannula in the right internal jugular vein. VA: drainage cannula through right femoral vein, return cannula in the right femoral artery

Given that conservative therapy for acute respiratory distress syndrome (ARDS) failed, the decision was to institute extracorporeal membrane oxygenation (ECMO). Since no obvious signs of low cardiac output were present, the decision was made to institute veno-venous (VV) ECMO, via a 25-French multi-orifice left femoral vein drainage cannula with its tip at the left iliac vein, and a 21-French right internal jugular vein return cannula with its tip at the right atrium, reaching flows of 5–6 l/minute (Fig. 2a). The patient was initially placed on sweep flow rate of 10 l/minute and FiO2 100% (PaO2 344 mmHg). The prior hemodialysis catheter was left for administration of medications and blood products, and the CVVHD was run through the VV-ECMO circuit. The patient was continued on lung protective ventilation with tidal volumes at 6 ml/kg (ideal body weight), PEEP 10cmH20 and FiO2 100% and the inhaled epoprostenol therapy was discontinued.

Three days later, the patient severe hypoxemia persists notwithstanding VV-ECMO at maximum settings (nadir PaO2 52 mmHg, arterial O2 saturation 81%). Oxygenator issues were ruled out by excluding any obvious thrombus in circuit and by confirming post-oxygenator high oxygenation levels (post-ECMO PaO2 332 mmHg). Nitric oxygen (NO) therapy was initiated to assist with right ventricular and pulmonary artery pressure off-loading. A supplementary 21-French drainage cannula was placed in the right femoral vein and its tip at the junction of the inferior vena cava and the right atrium (Fig. 2b), achieving flows of 7–8 L/min (veno-veno-venous [VV-V] ECMO). The patient oxygenation status intermittently improved (PaO2 310 mmHg), although a significantly elevated lactate dehydrogenase (LDH) was noticed (> 12,500 unit/L; normal range 313–618 units/L). Liver function panel was normal.

On next day, hypoxemia persists (PaO2 66 mmHg), with associated systemic hypotension (mean arterial pressure [MAP] of 52 mmHg) and signs of worsening tissue oxygenation (serum lactic acid 5.5 mmol/L). The team decided to switch to VV and VA ECMO with parallel circuits, in order to provide further cardiovascular support, relieve the patient’s refractory hypoxemia and provide adequate flows for his BSA.

The original VV-ECMO circuit consisting of a left femoral vein drainage cannula and right internal jugular vein return cannula was continued, and VA-ECMO was instituted through the existing venous cannula in the right femoral vein (which became the drainage cannula) and an additional 17-French right femoral artery return cannula with its tip at the distal aorta before its bifurcation (Fig. 2c). Distal right lower limb infusion cannula was placed to the right superficial femoral artery. No significant drop in the venous pressures in the original VV-ECMO or in the CVVHD lines was noticed when the four cannulations were instituted. The total flow of both circuits was 10–11 L/min: 5-6 L/min for the VV-ECMO and 5 L/min for the VA-ECMO.

The patient’s hemodynamics and oxygenation subsequently improved over the course of the next days (average MAP 68 mmHg, average PaO2 170 mmHg). A significant decrease in the LDH levels was noticed. No clinical or laboratory signs of upper body hypoxia (Harlequin Syndrome) was noticed. Adequate weaning of flow sweeps and FiO2 in both circuits was achieved. VV-ECMO was removed by hospital day #29. TEE demonstrated an improved left ventricular function (ejection fraction 45%) with persistent right ventricular dilation, for which the VA-ECMO was continued to provide further relief of the right ventricle and successfully removed 5 days later. The patient continued further ventilator weaning and planned for long-term acute care recovery.

## Discussion and conclusions

The complexity of ECMO continues to evolve to accommodate the increasingly challenging population of patients requiring extracorporeal support [10]. Several combinations of *“Hybrid”* ECMO configurations have been described [11]. The principle behind these *“hybrid”* configurations is that all cannulations, either separated or merged, drain into single ECMO device and subsequently, either separated or merged return to the patient [9, 12]. Parallel circuits represent a concept in the area of Extracorporeal Life Support. Malik et al. described the use of parallel VV and VV ECMO circuits for a patient with refractory hypoxemia and high cardiac output with the rationale that higher flows were necessary to handle the patient’s oxygenation status [5]. The case presented illustrates the utility of a parallel VV/VA ECMO in a patient with refractory hypoxic respiratory failure, circulatory failure with concomitant high cardiac output requirements. Initial VV-ECMO was started after maximal conservative strategies for ARDS failed. The persistent hypoxemia during VV-ECMO was attributed to insufficient flows to compensate for the patient’s large BSA, since gas exchange depends on blood flow calculated at 3 ml/kg/min in adults [13]. Therefore, an additional drainage cannula was placed (*“hybrid”* VV-V ECMO). VA-ECMO was not contemplated as the time of initial ECMO cannulation as the patient had no signs of reduced cardiac output.

Upon initiation of hybrid VV-V ECMO, the introduction of high return flows (7–8 L/min) unmasked pre-existent compensated right ventricular dysfunction and led to acute cardiovascular collapse. No obvious reason for a pre-existent right ventricular weakness could be pointed out. A possible underlying component of chronic pulmonary hypertension due to uncontrolled obstructive sleep apnea/obesity hypoventilation syndrome may explain this finding. Subsequently, the justification to implement a two-parallel circuit VV and VA ECMO was based on the following reasons: first, an additional hybrid arterial cannula (V-VA) incorporated in one circuit, would not be sufficient to provide adequate flows for both oxygenation and the cardiac output support given the patient’s BSA (total flows required was estimated at 10-11 L/min). Second, given that very high ECMO flows are associated with a higher shear stress and hemolysis [14], distribution of ECMO flows among two circuits would relieve this problem as this was demonstrated by a significant decreased in the LDH levels when switching to VV/VA ECMO. Third, the patient already had developed acute kidney injury requiring CVVHD, which was connected to the VV-ECMO circuit. High ECMO flows may be associated to high pressures in the CVVHD circuit, impairing the appropriate functioning of the dialysis [15], thus decreasing the velocity of flows going through the VV-ECMO circuit would avoid this problem.

While generally a percutaneous right ventricular assist device may be considered in such circumstances, in our patient it was not an option due to rapid development of profound hemodynamic instability and inability to use a fluoroscopy machine adjustable to the patient’s bed specifications to assist with the insertion of the device. Besides the commonly cited risks derived from the use of ECMO devices (e.g. bleeding, thrombosis, cannula malpositioning and migration, mixing phenomena, intra-cranial bleeding) [13], the presence of two parallel circuits may be associated to increased vascular injuries secondary to additional cannulation sites, higher nursing care, avoidance of early mobilization, higher institutional costs and longer intensive care unit stay.

The use of hybrid VV/VA ECMO circuit in parallel may be considered in patients with concomitant refractory hypoxemia, cardiogenic shock and high flow requirements, unattainable with conventional ECMO configurations. Being this an extremely resource intensive therapeutic option, institutions must be highly selective in the decision to pursue this strategy.

## Data Availability

Not applicable.

## Abbreviations

ECMO: Extracorporeal Membrane Oxygenation
ARDS: Acute Respiratory Distress Syndrome
VA: Veno-Arterial
V-V: Veno-Venous
BMI: Body Mass Index
BSA: Body Surface Area
PaO2: Partial Pressure of Arterial Oxygen
PaCO2: Partial Pressure of Arterial Carbon Dioxide
ICU: Intensive Care Unit
FiO2: Fraction of Inspired Oxygen
PEEP: Positive End-Expiratory Pressure
MAP: Mean Arterial Pressure
CVVHD: Continuous Veno-Venous Hemodialysis
TEE: Transesophageal Echocardiogram
NO: Nitric oxygen
LDH: Lactate Dehydrogenase (LDH)
ELSO: Extracorporeal Life Support Organization
